# Comparative Antigenicity and Pathogenicity of Two Distinct Genotypes of Highly Pathogenic Avian Influenza Viruses (H5N8) From Wild Birds in China, 2020–2021

**DOI:** 10.3389/fmicb.2022.893253

**Published:** 2022-04-27

**Authors:** Wenming Jiang, Shuo Liu, Xin Yin, Zhixin Li, Zouran Lan, Luosong Xire, Zhongbing Wang, Yinqian Xie, Cheng Peng, Jinping Li, Guangyu Hou, Xiaohui Yu, Rongzhao Sun, Hualei Liu

**Affiliations:** ^1^China Animal Health and Epidemiology Center, Qingdao, China; ^2^Ningxia Hui Autonomous Region Animal Disease Prevention and Control Center, Yinchuan, China; ^3^Shandong Provincial Center for Animal Disease Control, Jinan, China; ^4^Tibet Autonomous Region Veterinary Biological Pharmaceuticals Factory, Lhasa, China; ^5^Shanxi Animal Disease Prevention and Control Center, Taiyuan, China; ^6^Shaanxi Animal Disease Prevention and Control Center, Xi’an, China

**Keywords:** H5N8, highly pathogenic avian influenza, wild birds, genetic, pathogenicity, antigenicity

## Abstract

To date, there have been three epidemic waves of H5N8 avian influenza worldwide. The current third epidemic wave began in October 2020 and has expanded to at least 46 countries. Active and passive surveillance were conducted to monitor H5N8 viruses from wild birds in China. Genetic analysis of 10 H5N8 viruses isolated from wild birds identified two different genotypes. Animal challenge experiments indicated that the H5N8 isolates are highly pathogenic in chickens, mildly pathogenic in ducks, while pathogenicity varied in BALB/c mice. Moreover, there were significant differences in antigenicity as compared to Re-11 vaccine strain and vaccinated chickens were not completely protected against challenge with the high dose of H5N8 virus. With the use of the new matched vaccine and increased poultry immune density, surveillance should be intensified to monitor the emergence of mutant strains and potential worldwide spread *via* wild birds.

## Introduction

The highly pathogenic H5N1 avian influenza virus (A/goose/Guangdong/1/1996(H5N1); clade 0) emerged from China in 1996 (Xu et al., 1999) and has spread throughout Eurasia and Africa possibly *via* migratory bird paths and have evolved into various genetic and antigenic clades and subclades since 2003 (WHO/OIE/FAO H5N1 Evolution Working Group, 2012; Li et al., 2020). These viruses have caused huge economic losses to the poultry industry and pose a substantial threat to human health. Since 2010, clade 2.3.4 H5 viruses have evolved and have gradually become dominant globally.

H5N8 avian influenza virus (AIV) clade 2.3.4 was first detected from a domestic duck in China, in 2010 (Wu et al., 2014; Li et al., 2016). To date, there have been three epidemic waves of H5N8 viruses worldwide. The first epidemic wave of H5N8 began in early 2014 in domestic and wild birds in South Korea and Japan and spread to Russia, several European countries, and the United States by 2015 (Lee et al., 2014, 2015, 2016; Wu et al., 2014; Saito et al., 2015; The Global Consortium for H5N8 and Related Influenza Viruses, 2016). Additionally, an H5N2 virus containing gene segments related to H5N8 was identified in Canada in late 2014 (Pasick et al., 2015). Bayesian phylogenetic analysis revealed that H5Ny lineages were introduced into North America from Eurasia and into South Korea from Europe likely through migratory waterfowl (The Global Consortium for H5N8 and Related Influenza Viruses, 2016; Baek et al., 2021).

The second epidemic wave started in late 2016 and lasted until early 2017 (Beerens et al., 2017; Fusaro et al., 2017; Kim et al., 2017; Pohlmann et al., 2017; Selim et al., 2017; Wade et al., 2018; Yehia et al., 2018). H5N8 AIVs belonging to clade 2.3.4.4 reemerged in Europe, Asia, and Africa, suggesting that clade 2.3.4 H5 AIVs, particularly the H5N8 subtype, have a propensity for rapid global spread in migratory birds (Li et al., 2017; Napp et al., 2018).

The current third epidemic wave began in October 2020 and had the highest number of outbreaks. Outbreaks of H5N8 in poultry and wild birds have been reported in several European countries, including Denmark, Germany, Ireland, the Netherlands, and the United Kingdom (Lewis et al., 2021). Meanwhile, H5N8 outbreaks have been reported in poultry and wild birds in the Middle East (Israel) and East Asia (Japan, South Korea, and China), and have expanded to at least 46 countries (Isoda et al., 2020; Baek et al., 2021; Li et al., 2021; Sakuma et al., 2021). The third wave is more serious than the second in terms of the number of cases and extent of spread. To date, more than 20 million poultry have been slaughtered in South Korea and Japan due to infection with AIVs belonging to clade 2.3.4.4b, as determined by phylogenetic analysis (Twabela et al., 2020).

H5 viruses are known to infect humans. To date, 863 laboratory-confirmed human cases of H5N1 infection have been reported to the World Health Organization, which resulted in 456 (52.8%) deaths. Surprisingly, the first human cases of H5N8 infection were reported in Russia in December 2020 (Shi and Gao, 2021). Seven poultry farm workers who participated in a response operation to contain an outbreak of H5N8 tested positive, suggesting the potential for human infection.

From October 2020 to June 2021, several outbreaks of H5N8 in wild birds were reported in China. Therefore, the aim of the present study was to investigate the genetic, pathogenic, and antigenic characteristic of H5N8 by active and passive surveillance in China.

## Materials and Methods

### Ethics Statements and Facility

The study protocol was approved by the Ethics Committee of China Animal Health and Epidemiology Center (Qingdao, China). All experiments with lethal H5 viruses were performed in a biosafety level 3 facility, and all animal experiments were performed in high-efficiency particulate air-filtered isolators at the China Animal Health and Epidemiology Center. All experimental animals were humanely handled in accordance with animal welfare.

### Sampling

From September 2020 to June 2021, persistent surveillance of AIV infection in wild birds in China was conducted by collecting swab samples from the tracheae and cloacae of different species of wild birds and liver and lung tissue samples from dead birds. The swab samples were placed in transport medium (penicillin [2,000 IU/ml], streptomycin [2 mg/ml], amikacin [1,000 IU/ml], nystatin [2,000 IU/ml], and 10% glycerol [v/v] in sterile phosphate-buffered saline [pH 7.2]), transported to our laboratory at 4°C within 72 h, and stored at −70°C.

### Virus Isolation and Identification

For virus isolation, the tissue samples were homogenated in transport medium followed by three freeze–thaw cycle. The homogenated tissue samples and swab samples were clarified by centrifugation at 10,000 × *g* for 5 min at 4°C, and the supernatants were inoculated into the allantoic cavity of 10-day-old specific-pathogen-free (SPF) chicken embryos, which were then incubated at 37°C for 4 days and checked daily. Dead embryos were removed and stored at 4°C. After the incubation period, live embryos were sacrificed at 4°C and the allantoic fluid was collected for testing with the hemagglutination assay.

RNA was extracted from the allantoic fluid of embryonated eggs with the QIAamp Viral RNA Mini Kit (Qiagen GmbH, Hilden, Germany) and the HA gene was amplified using the primer pair 5´-AGTGAARTGGAATATGGYMACTG-3′/5´-AACTGAGTGTTCATTTTGTCAAT-3′. Positive amplicons were confirmed by sequencing. H5 viruses grown in 10-day-old SPF embryonated eggs were purified by three rounds of the limited dilution method.

Subsequently, the complete genomes of purified H5 viruses were amplified using the PrimeScript One-step RT-PCR Kit (Takara Bio, Inc., Shiga, Japan) as described previously (Hoffmann et al., 2001; Jiang et al., 2017). The PCR products were purified with the QIAquick PCR purification kit (Qiagen GmbH) and sequenced using the ABI 3730xl DNA Analyzer (Applied Biosystems, Carlsbad, CA, United States). The nucleotide sequences were edited using the SeqMan module of the DNASTAR® Lasergene® package (DNASTAR, Inc., Madison, WI, United States). Phylogenetic analyses were conducted with Molecular Evolutionary Genetics Analysis (MEGA) software ver. 5.10^1^ and aligned using the ClustalW algorithm.^2^ Phylogenetic trees were constructed using the neighbor-joining method with a bootstrap value of 1,000; 96% sequence identity cutoffs were used to categorize the groups of each gene segment in the phylogenetic trees. The bioinformatics data were also analyzed based on the genome sequences.

### Studies in Chickens

Two isolates with different genotypes (SX1/2020 and NX18/2020) were selected for pathogenicity studies in chickens, ducks, and mice.

To assess the pathogenicity of the SX1/2020 and NX18/2020 viruses in chickens, the intravenous pathogenicity index (IVPI) was determined in accordance with the recommendations of the World Organization for Animal Health. Six-week-old SPF chickens (*n* = 10/group) were intravenously inoculated with 0.1 ml of a 1/10 dilution of bacteria-free fresh infectious allantoic fluid containing the challenge virus (HA titer > 16). 10 chickens were inoculated with 0.01 M phosphate-buffered saline as a control group. The chickens were then examined daily for 10 days for signs of disease or death. At each observation, each chicken was scored based on a 4-point scale (0, normal, 1, sick; 2, severely sick; or 3, dead). The IVPI was calculated as the mean score of each chicken at each observation point over the 10-day period.

10 additional chickens were inoculated intranasally with 10^6^ 50% egg infective dose (EID_50_) of each virus in a 0.1-mL volume. At 3 days post-infection (dpi), the chickens were subjected to euthanasia and necropsy. The brain, heart, liver, spleen, lungs, intestine, and kidneys were collected for detection of viral RNA. Tracheal and cloacal swabs were collected from all birds for detection of virus shedding.

### Studies in Ducks

To assess the pathogenicity of the SX1/2020 and NX18/2020 viruses in ducks, 3-week-old SPF ducks (*n* = 10/group) were intranasally inoculated with 0.1 ml of allantoic fluid containing 10^6^ EID_50_ of the tested virus. The ducks were then observed for signs of disease or death until 14 dpi. At necropsy (3 dpi), the brain, heart, liver, spleen, lungs, intestine, and kidneys of five ducks were collected for detection of viral RNA. Tracheal and cloacal swabs were collected from the remaining birds at 3 dpi for detection of virus shedding. At the end of the experiment, the animals were subjected to blood collection for assessment of seroconversion, followed by euthanasia.

### Studies in Mice

To assess the pathogenicity of the SX1/2020 and NX18/2020 viruses in a mammalian host, 6-week-old female Balb/c mice (Charles River Laboratories, Beijing, China; *n* = 14/group) were lightly anesthetized with CO_2_ and inoculated intranasally with 10^6^ EID_50_ of each virus in a volume of 50 μl. Nine of the 14 mice were euthanized at 3, 4, and 5 dpi for virus titration of the nasal turbinate, lungs, spleen, brain, and liver. The remaining five mice were monitored daily for weight loss and mortality until 14 dpi. At the endpoints of the experiment, the mice were anesthetized and sacrificed humanely.

### Antigenic Analyses

Antigenic analyses were performed using cross-hemagglutination inhibition (HI) tests with polyclonal antisera against the indicated viruses. To generate the antisera, 21-day-old SPF chickens were injected with 1 ml of oil emulsion-inactivated vaccines derived from the selected viruses and sera samples were collected at 21 dpi. Antibodies to HI were tested with 0.5% (v/v) chicken erythrocytes.

### Generation of Recombinant Viruses

A recombinant virus (rSD18) was generated with a reverse-genetics method using eight bidirectional pHW2000 plasmids. Human embryonic kidney 293 T cells were co-transfected with 0.8 μg of each of the six bidirectional pHW plasmids (corresponding to PB2, PB1, PA, NP, M, and NS) as well as the HA and NA genes of the SD18/2020 virus. The pHW plasmids expressing six internal genes were all derived from the A/PR/8/34(H1N1) virus with Lipofectamine 3,000 transfection reagent (Life Technologies, Carlsbad, CA, United States). The HA sequence of the SD18/2020 virus was attenuated from PLREKRRKRG to PLRETRG by deletion of the multibasic amino acid motif at the HA cleavage site. After 24 h, the cells were treated with L-(tosylamido-2-phenyl) ethyl chloromethyl ketone-treated trypsin (Sigma–Aldrich Corporation, St. Louis, MO, United States) at a final concentration of 2 μg/ml. After 72 h, the supernatants of transfected cells were collected and used to inoculate 10-day-old SPF chicken embryos, which were then incubated at 37°C for 72 h. Vaccine batches were produced in SPF chicken embryos after five egg passages of recombinants.

### Vaccination and Challenge Study in Chickens

Two oil-adjuvant whole-virus inactivated vaccines were prepared from Re-11 [the HA and NA gene donor (A/duck/Guizhou/S4184/2017, H5N6), HA gene clade (clade 2.3.4.4 h)] (Zeng et al., 2020) and rSD18 (the inactivated virus mixed with mineral oil adjuvant at 1:2 (v/v) and then emulsified) by Qingdao Yebio Bioengineering Co., Ltd. (Qingdao, China). Groups of three-week-old SPF chickens were vaccinated with the Re-11 and rSD18 vaccines. 3 weeks later, the chickens were intranasally challenged with 10^6^ EID_50_ of the tested viruses. Tracheal and cloacal swabs were collected from the chickens for virus titration at 3 and 5 dpi, and clinical signs were monitored daily until 10 dpi. In addition, two groups of 10 chickens were used as challenge controls. At the end of the experiment, the animals were anesthetized and sacrificed humanely.

## Results

### Molecular and Phylogenetic Analysis

**Figure 1:**
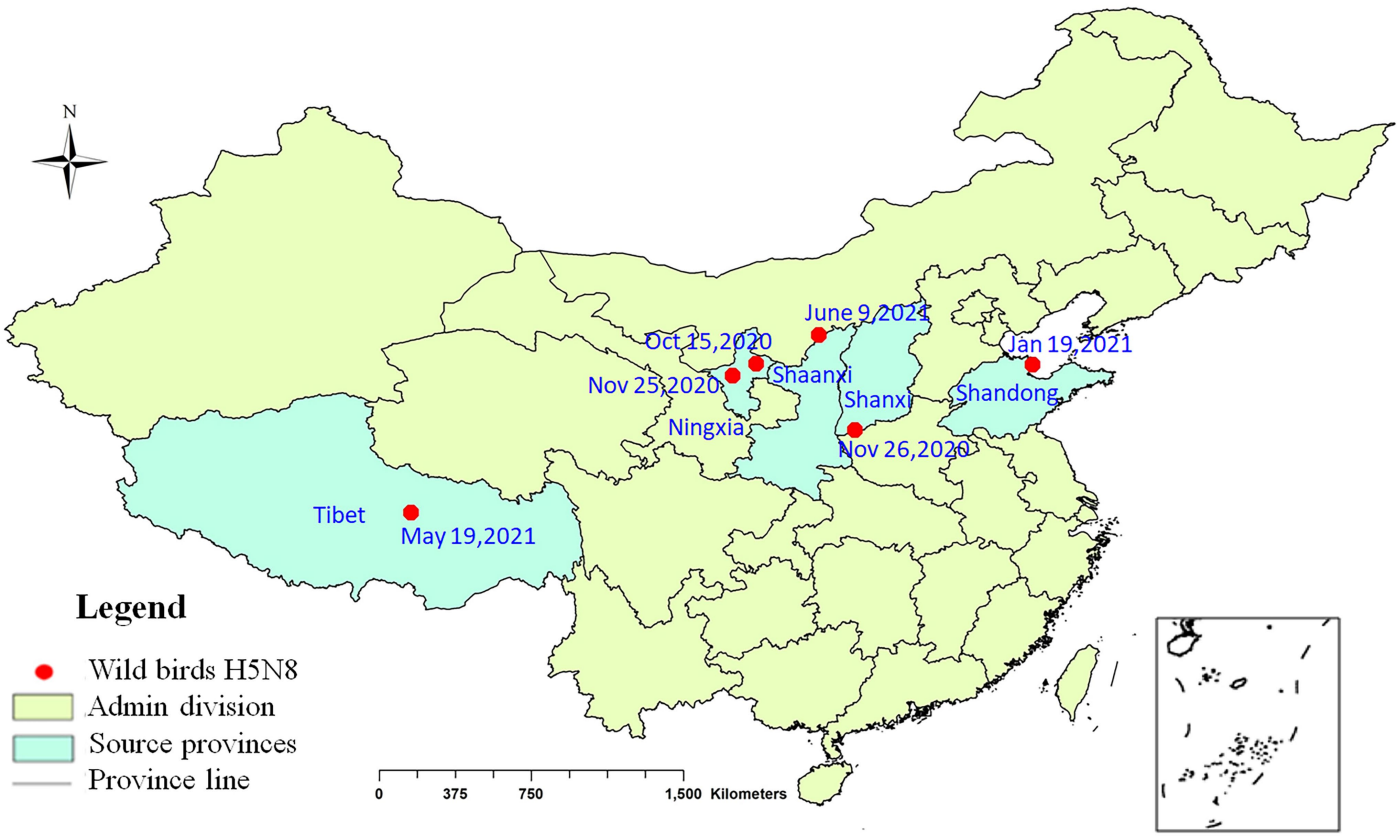
Geographical distribution of H5N8 avian influenza viruses detected in wild birds in China, 2020–2021. Red dot indicates sampling sites. Dates refer to the day of initial H5N8 virus isolated in wild birds in each site.

During September 2020 to June 2021, 10 viruses were isolated from eight kinds of wild birds in Shanxi, Ningxia, Shandong, Tibet, and Shaanxi provinces (Figure 1). The information of the strains is shown in Supplementary Table S1.

**Figure 2:**
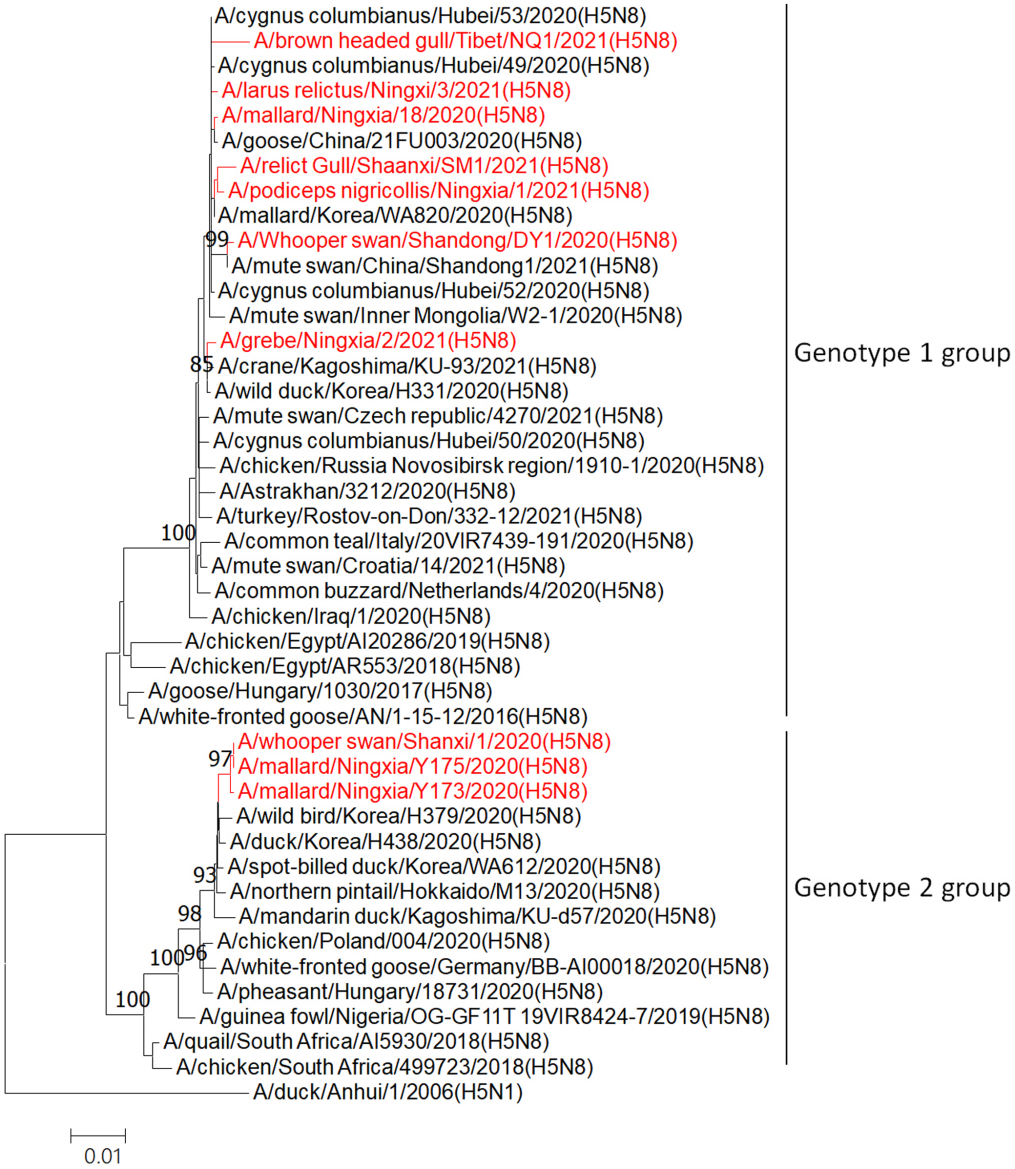
Phylogenetic analyses of the HA genes of H5N8 highly pathogenic AIVs. Phylogenetic trees were constructed with MEGA (ver. 5.10) software using the neighbor-joining method. Bootstrap analysis was performed with 1,000 replications. The viruses sequenced in this study are shown in red in the phylogenetic trees. Scale bars indicate nucleotide substitutions per site.

Phylogenetic analysis of HA genes showed that all 10 viruses isolated from wild birds in this study belonged to clade 2.3.4.4b during September 2020 to June 2021 (Figure 2). Furthermore, on the basis of their genomic similarity and phylogenetic analysis of genome sequences (Supplementary Figures S1–S7), the 10 H5N8 HPAIVs in this study were divided into two genotypes: seven strains belong to the genotype 1 and three strains formed a different genotype 2.

The HA genes of clade 2.3.4.4b viruses contained a series of basic amino acids (PLREKRRKR/G) at the cleavage sites, a signature of highly pathogenic avian influenza virus The receptor binding sites of the viral HA genes possessed the residues Q226 and G228 (H3 numbering), suggesting preferential binding to avian-like receptors (Tharakaraman et al., 2013). However, the receptor binding site mutations A137, N158, A160, N186, I192, Q222, and R227 (H3 numbering) could increase binding to SAα-2,6Gal human-like receptors (Yang et al., 2007; Gao et al., 2009; Wang et al., 2010; Guo et al., 2017).

Bioinformatics analysis identified many mutations that would increase virulence in mice, such as R114 and I115 (H3 numbering) of the HA gene (Wessels et al., 2018); D30, M43, and A215 of the M1 gene (Fan et al., 2009; Nao et al., 2015); S42, E55, E66 (SX1/2020, Y173/2020, and Y175/2020), M106, and F138 (except SX1/2020, Y173/2020, and Y175/2020) of the NS1 gene (Jiao et al., 2008; Ayllon et al., 2014; Li et al., 2018); the NS1 C-terminal ESEV motif of the PDZ domain at position aa227–230 (Jackson et al., 2008; Soubies et al., 2010; Zielecki et al., 2010); combination of V89, D309, K339, G477, V495, E627, and T676 of the PB2 gene (Li et al., 2009); V3 (except NQ1/2021) and G622 of the PB1 gene (Feng et al., 2016; Elgendy et al., 2017); and D383 of the PA gene (Song et al., 2015; Suttie et al., 2019).

### Pathogenicity in Chickens

To assess pathogenicity in chickens, 6-week-old SPF chickens were inoculated with viruses to determine the IVPI. Both SX1/2020 and NX18/2020 viruses caused 100% mortality within 1 day, conferring an IVPI of 3.00 and indicating that both were highly pathogenic in chickens (Table 1).

**Table 1:** Replication and virulence of 2.3.4.4b H5N8 viruses in chickens and ducks.

Avian species	Viruses	IVPI	Virus shedding on day 3 post inoculation: positive/total		Virus replication in organs on day 3 p.i.: positive/total						Death/Total	Sero-conversion: positive/total
			Pharynx	Cloacae	Lung	Intestine	Liver	Spleen	Kidney	Brain		
Chicken	SX1/2020	3.00	10/10	10/10	10/10	10/10	10/10	10/10	10/10	10/10	10/10	/
	NX18/2020	3.00	10/10	10/10	10/10	10/10	10/10	10/10	10/10	10/10	10/10	/
Duck	SX1/2020	/	10/10	10/10	5/5	5/5	4/5	3/5	5/5	3/5	1/5	4/4
	NX18/2020	/	10/10	10/10	5/5	5/5	4/5	4/5	5/5	4/5	2/5	3/3

*The intravenous pathogenicity index (IVPI) test was carried out according to the OIE (World Organisation for Animal Health) manual. In brief, ten 6-week-old SPF chickens were intravenously inoculated with 0.1 ml of a 1/10 dilution of the fresh infectious allantoic fluid (HA titer > 16) and ten chickens were inoculated with 0.01 M PBS as a control group. All of the chickens were examined daily for 10 days. At each observation, each chicken was scored based on the condition: 0 (normal), 1 (sick), 2 (severely sick), and 3 (dead). IVPI was the mean score per chicken per observation over the ten-day period*. *Groups of 15 three-week-old specific-pathogen-free ducks were inoculated i.n. with 10^6^ EID_50_ of each virus in a 0.1-ml volume. Pharyngeal and cloacal swabs were collected from all birds on Day 3 p.i., and then five birds in each group were euthanized, and their organs were detected for H5N8 viruses by RT-PCR. The remaining ten ducks in each group were observed for two weeks*.

All chickens infected intranasally with 10^6^ EID_50_ of the SX1/2020 and NX18/2020 viruses died within 5 dpi. Both viruses were detected in the heart, liver, spleen, lungs, kidneys, intestine, and brain samples collected during necropsy of inoculated chickens at 3 dpi. Virus shedding was detected from the tracheal and cloacal swabs of all dead chickens inoculated with the SX1/2020 and NX18/2020 viruses at 3 dpi (Table 1).

### Pathogenicity in Ducks

To assess pathogenicity in ducks, 3-week-old SPF ducks were inoculated with the SX1/2020 and NX18/2020 viruses, which caused 20%—40% mortality within 14 dpi, indicating that both were moderately pathogenic in ducks.

To investigate the replication of these viruses in ducks, five ducks from each group were euthanized at 3 dpi to assess the viral load in the heart, liver, spleen, lungs, kidneys, intestine, and brain. The remaining five ducks in each group were assessed for seroconversion (Table 1).

Both viruses were detected in the heart, liver, spleen, lungs, kidneys, intestine, and brain samples collected during necropsy of inoculated ducks at 3 dpi. All ducks inoculated with the SX1/2020 and NX18/2020 viruses exhibited virus shedding and seroconversion (Table 1). The results indicated that both viruses were moderately pathogenic in ducks.

### Replication and Pathogenicity in Mice

To assess the replication and virulence of the SX1/2020 and NX18/2020 viruses in a mammalian host, 6-weeks-old Balb/c mice (*n* = 14/group) were inoculated intranasally with 10^6^ EID_50_ of each virus. On 3, 4, and 5 dpi, three mice from each group were euthanized to measure the viral load in nasal turbinate, lungs, spleen, brain, and liver, while the other five were observed for body weight changes and death until 14 dpi.

**Figure 3:**
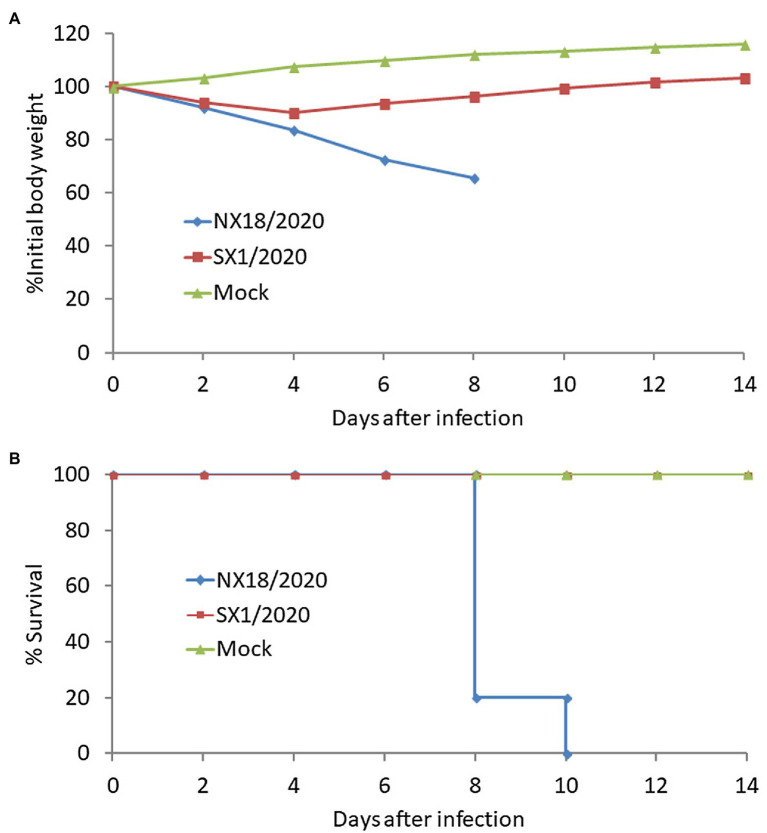
Virulence of the H5N8 viruses in Balb/c mice intranasally inoculated with a 10^6^ EID_50_ of virus. Body weight changes **(A)** and mortality **(B)** of Balb/c mice were monitored daily for 14 days.

At 3, 4, and 5 dpi, mice infected with the NX18/2020 virus had high titers in the nasal turbinate, lungs, spleen, and brain, but not the liver (Table 2). The mice exhibited progressive signs of infection, such as inactivity, ruffled fur, lack of appetite, hunched backs, and labored breathing. The body weights of mock-infected mice gradually increased from 1 to 14 dpi. In contrast, the infected mice experienced dramatic weight loss of more than 25% and all died within 8 dpi (Figure 3). These results suggest that the NX18/2020 virus not only effectively replicated in the nasal turbinate, lung, spleen, and brain without preadaptation, but was also lethal.

**Table 2:** Replication level of SX1/2020 and NX18/2020 strains in organs of experimentally infected Balb/c mice.

Strains	Virus titers in organs of experimentally infected mice (log_10_ EID_50_/g)^a^			
	Tissue	3 day	4 day	5 day
SX1/2020	Nasal turbinate	3.86 ± 0.32	3.83 ± 0.28	4.12 ± 0.45
	Lung	3.67 ± 0.36	3.82 ± 0.37	4.03 ± 0.42
	Liver	–	–	–
	Spleen	–	–	–
	Brain	–	–	–
NX18/2020	Nasal turbinate	4.45 ± 0.33	4.78 ± 0.32	5.32 ± 0.46
	Lung	4.27 ± 0.38	4.56 ± 0.38	5.18 ± 0.45
	Liver	–	–	–
	Spleen	3.07 ± 0.30	3.16 ± 0.31	3.46 ± 0.33
	Brain	2.23 ± 0.22	2.92 ± 0.26	3.26 ± 0.35

a *Groups of fourteen female Balb/c mice were intranasally inoculated with a 10^6^ EID_50_ of each virus. On 3, 4, and 5 dpi, three mice of each group were euthanatized after anesthetization, and the nasal turbinates, brains, lungs, livers, and spleens of the mice of each group were pooled separately and homogenized in L-15 medium containing antibiotics to make a 10% w/v tissue homogenate for virus titration in embryonated chicken eggs. Values are mean ± SD. –, virus titer lower than the detection limit*.

In contrast, the SX1/2020 virus replicated poorly and was only detected in the nasal turbinate and lungs of mice and was not fatal (Figure 3), indicating that H5N8 viruses circulating in nature have different pathotypes in mice.

### Antigenic Analyses

Since December 2018, the inactivated reassortant vaccine Re-11 has been extensively used to control the spread of clade 2.3.4.4 viruses in China. To determine whether antigenic drift had occurred, antigenicity of the H5N8 viruses was evaluated with the cross-HI assay.

The results showed that the H5N8 isolates weakly reacted with Re-11 antisera (Supplementary Table S2). The cross-reactive HI titers of the Re-11 antiserum against the H5N8 isolates were 32- to 128-fold lower than that against the homologous Re-11 antigen (9 log2). In contrast, the cross-reactive HI titers of the antiserum against the Re-11 antigen from the H5N8 viruses were also 32- to 128-fold lower than that against the homologous H5N8 isolates. The amino acid homology between these isolates and Re-11 was 91.2–91.7%. These results indicate that the H5N8 viruses exhibited severe antigenic drift as compared to the more established H5 vaccine strains.

### Protective Efficacy of the Vaccines

To determine whether these isolates reduce the protective effect of commercially available vaccines *in vivo*, the protection efficacies of the inactivated reassortant H5N1/PR8 vaccine Re-11 and rSD18 vaccine were evaluated.

During the 10-day observation period, birds vaccinated with the Re-11 vaccine displayed no clinical signs and all survived. In addition, the tracheal and cloacal swabs from only 10.0–20.0% of the experimental chickens exhibited virus shedding at 3 and 5 dpi. The rSD18-vaccinated birds all survived and displayed no clinical signs of infection. In addition, no virus shedding was detected in tracheal or cloacal swabs from any of the experimental chickens at 3 and 5 dpi (Table 3). These results suggest that the Re-11 vaccine did not provide complete protection against the high dose of antigenically distinct highly pathogenic H5N8 AIV.

**Table 3:** Protection efficacy of H5 Re-11 vaccine against SX1/2020 and NX18/2020 in chickens^a^.

		Challenge test results, by swab type, no. positive birds/no.tested (mean titer ± SD)^b^						No.surviving
Group	Vaccine	HI titer ± SD, log2		3 dpi		5 dpi		birds/total no.
		Re-11	Isolate	Tracheal	Cloacal	Tracheal	Cloacal	
SX1/2020	Re-11	10.6 ± 0.42	5.2 ± 0.25	1/10 (3.52)	1/10 (3.62)	1/10 (3.46)	1/10 (3.85)	10/10
	rSD18	5.6 ± 0.30	10.3 ± 0.37	0/10	0/10	0/10	0/10	10/10
	Mock	ND	ND	4/4 (3.98 ± 1.03)	4/4 (4.12 ± 1.35)	ND	ND	0/10
NX18/2020	Re-11	10.6 ± 0.38	5.2 ± 0.22	2/10 (3.65 ± 1.06)	2/10 (3.78 ± 1.14)	2/10 (3.58 ± 1.06)	2/10 (3.87 ± 1.25)	10/10
	rSD18	5.1 ± 0.32	10.2 ± 0.39	0/10	0/10	0/10	0/10	10/10
	Mock	ND	ND	4/4 (3.87 ± 1.15)	4/4 (4.25 ± 1.35)	ND	ND	0/10

a *Chickens were immunized with the Re-11 vaccine, and HI antibody titers were determined on day 21 post-vaccination. HI, Hemagglutination inhibition assay; dpi, days post-infection; ND, not done*.

b *Chickens were challenged with 10^6^ 50% egg infectious dose (EID_50_) of SX1/2020 or NX18/2020 virus; virus titers are expressed as log10 EID_50_/0.1 ml*.

## Discussion

Since 2014, there have been three global waves of the H5N8 pandemic in wild birds and poultry. The spread of H5N8 viruses in many countries in Asia, Europe, Africa, and North America has resulted in numerous outbreaks in domestic poultry (Lee et al., 2014, 2015, 2016; Wu et al., 2014; Saito et al., 2015; The Global Consortium for H5N8 and Related Influenza Viruses, 2016). During the first and second epidemic waves, H5N8 strains were occasionally detected from wild birds, ducks, and geese in China, although the damage caused by these viruses was limited (Cui et al., 2020). However, the current third epidemic wave of H5N8 viruses has spread with a vengeance, resulting in heavy losses to the poultry industry worldwide.

In this study, positive and passive surveillance was conducted to monitor the invasion and spread of H5N8 viruses in China. The H5N8 viruses were first detected in two healthy mallards in Ningxia Province in October 2020 and dead whooper swans found in Shanxi Province in November 2020. Afterward, these viruses have been detected in seven other varieties of wild birds in several provinces. Genetic analysis revealed that two distinct genotypes of H5N8 viruses were circulating in wild birds in China, with wide spread of genotype 1. The results of the present study are consistent with those of previous reports (Baek et al., 2021; Cui et al., 2021). Interestingly, virusesw isolated from different individuals at the same site may be of different genotypes, which illustrates the complexity of viruses in China.

Animal studies have shown that H5N8 viruses are highly pathogenic in chickens, but cause relatively mild disease in ducks. In previous studies, some H5N8 strains could kill nearly 100 percent of the challenged ducks (Dinev et al., 2020; Koethe et al., 2020). The pathogenicity of the virus may depend on the strain and duck species. In practice, even with the adoption of biosecurity measures, the production of ducks has been poorer than that of chickens and other pathogens, such as *Escherichia coli* and *Riemerella anatipestiferis*, which cause higher mortality rates in ducks. Furthermore, there is a clear antigenic difference between H5N8 viruses and the H5N1 vaccine strain Re-11 used in China against 2.3.4.4 viruses, as vaccinated chickens are not completely protected against challenge with the high dose of H5N8 viruses. The results were inconsistent with those of Cui et al., which may be related to bird species and immune background where the layer chickens and ducks in the field were immunized with two or more doses of the H5/H7 AI inactivated vaccine (Cui et al., 2021). As we known, under field conditions, birds unlikely to get sustained high levels of antibody and would more likely be susceptible to infection and virus shedding. Normally, when HI titer to the challenge virus of H5 subtype is above 4 log2, the vaccinated chickens were completely protected without virus shedding. However, in this study, vaccinated chickens with HI antibody titers of 5.2 log2 still shed viruses after challenge. It is highly likely that the reduced immune protection is caused by the high dose of challenge virus. The 10^6^EID_50_ dose of the challenged H5N8 virus in this study is tenfold higher than the normal dose (10^5^EID_50_).

Animal studies have shown that the virulence of H5N8 viruses in mice varies among strains, as a highly pathogenic virus was lethal in mice without preadaptation and effectively replicated in several organs, while replication of a less pathogenic virus was limited in some organs. The H5N8 virus isolated in 2013 showed similar results (Wu et al., 2014).

In 2004 and 2005, an epidemic of the H5N1 virus occurred in China (Chen, 2009). Thus, the Chinese government adopted comprehensive prevention and control measures, including surveillance, culling, and mass vaccination, which achieved great success. For example, replacement of the Re-4 vaccine with the Re-7 vaccine in 2014 completely eliminated infection of a clade 7.2 virus (Liu et al., 2016). In 2017, the use of the H7N9-Re1 vaccine successfully controlled H7N9 infection in humans and highly pathogenic viruses in chickens (Shi et al., 2018; Jiang et al., 2019). However, vaccines were less effective in controlling other clades of the H5 virus, such as clade 2.3.4, which is found in chickens and especially waterfowl, while clade 7.2 and H7N9 viruses are found mainly in chickens and less often in waterfowl. In China, the number of waterfowl is huge and many are raised in open environments in contact with wild birds. Clade 2.3.4.4b viruses circulate in wild birds and spread easily to waterfowl and, thus, are more difficult to control. In addition, the immune density of waterfowl is much lower than that of chickens, which causes waterfowl to become more susceptible to infection and further spread of the virus. Due to continuous mutation and reassortment, antigenic drift of avian influenza viruses occurs frequently. Timely updating the vaccine seeds could maintain the effectiveness of the vaccine in control and elimination of the target avian influenza virus. Although the Re-11 vaccine strain was updated by antigenically matched Re-14 vaccine to prevent and control clade 2.3.4.4b viruses since 2022, many challenges remain. With the use of new matched vaccines and increased poultry immune density, surveillance should be strengthened to early detect the emergence of mutant strains and worldwide spread *via* wild birds.

## Data Availability Statement

The datasets presented in this study can be found in online repositories. The names of the repository/repositories and accession number(s) can be found in the article/Supplementary Material.

## Ethics Statement

The animal study was reviewed and approved by Ethics Committee of China Animal Health and Epidemiology Center.

## Author Contributions

WJ and HL: conceptualization and methodology. ZhL, ZoL, LX, ZW, and YX: sample collection. SL, XY, and CP: genetic analysis. WJ, HL, ZhL, ZoL, LX, ZW, and YX: investigation. XnY, XaY, and RS: data curation. WJ: writing—original draft preparation. HL: writing—review and editing. GH and JL: animal experiment. All authors contributed to the article and approved the submitted version.

## Funding

This work was supported by National Key Research and Development Program (2021YFD1800201), Natural Science Foundation of Ningxia Hui Autonomous Region (2022AAC02071), and Shandong Provincial Key Research and Development Program (2022CXGC010606).

## Conflict of Interest

The authors declare that the research was conducted in the absence of any commercial or financial relationships that could be construed as a potential conflict of interest.

## Publisher’s Note

All claims expressed in this article are solely those of the authors and do not necessarily represent those of their affiliated organizations, or those of the publisher, the editors and the reviewers. Any product that may be evaluated in this article, or claim that may be made by its manufacturer, is not guaranteed or endorsed by the publisher.

## Footnotes

^1^ http://www.megasoftware.net

^2^ http://www.clustal.org/

## Supplementary Material

The Supplementary Material for this article can be found online at: https://www.frontiersin.org/articles/10.3389/fmicb. 2022.893253/full#supplementary-material
