# Supplementary material for: Comparative Antigenicity and Pathogenicity of Two Distinct Genotypes of Highly Pathogenic Avian Influenza Viruses (H5N8) From Wild Birds in China, 2020–2021
Source: Front Microbiol. 2022 Apr 27;13:893253. doi: 10.3389/fmicb.2022.893253 (PMC9122345; doi:10.3389/fmicb.2022.893253)
Supplement: Supplementary file 1 [file Data_Sheet_1.docx]

Supplementary files for

Comparative antigenicity and pathogenicity of two distinct genotypes of highly pathogenic avian influenza viruses (H5N8) from wild birds in China, 2020–2021

Figures S1. Phylogenetic analyse of the PB2 gene of H5N8 highly pathogenic avian influenza viruses. Trees were constructed with MEGA5.10 software using the neighbor-joining method. Bootstrap analysis was performed with 1,000 replications. The viruses sequenced in this study are shown in red in the phylogenetic trees. Scale bars indicate nucleotide substitutions per site.


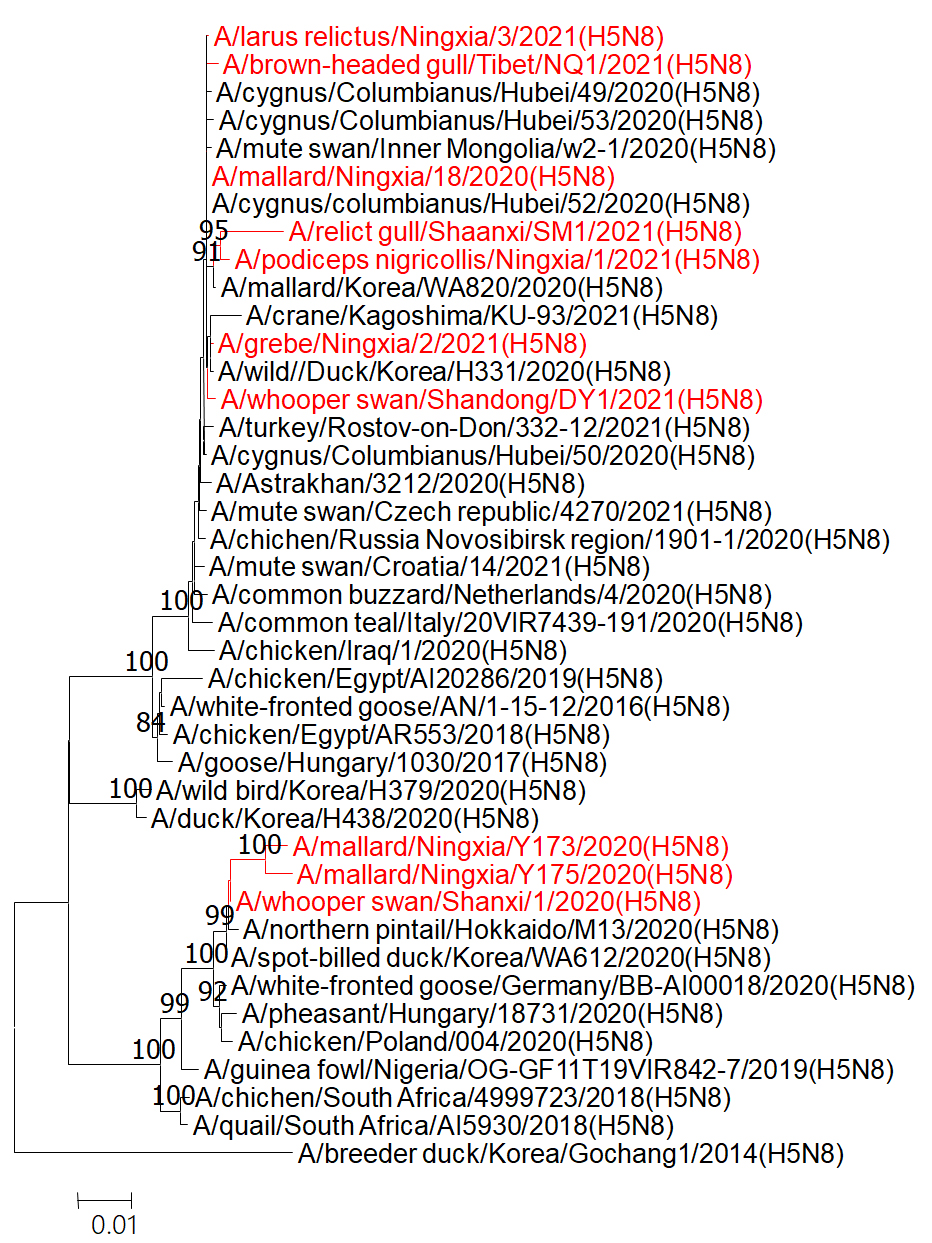


Figures S2. Phylogenetic analyse of the PB1 gene of H5N8 highly pathogenic avian influenza viruses. Trees were constructed with MEGA5.10 software using the neighbor-joining method. Bootstrap analysis was performed with 1,000 replications. The viruses sequenced in this study are shown in red in the phylogenetic trees. Scale bars indicate nucleotide substitutions per site.


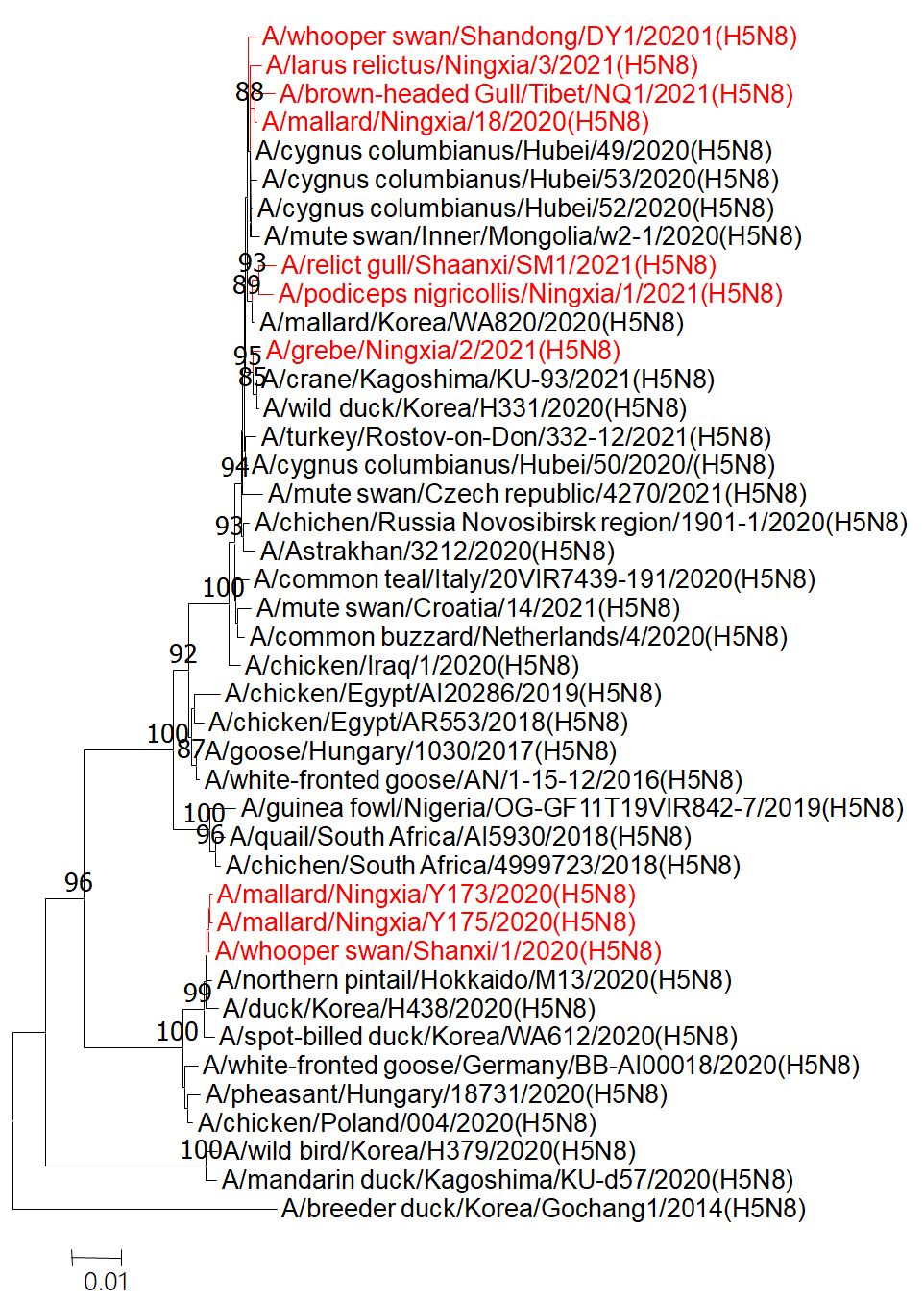


Figures S3. Phylogenetic analyse of the PA gene of H5N8 highly pathogenic avian influenza viruses. Trees were constructed with MEGA5.10 software using the neighbor-joining method. Bootstrap analysis was performed with 1,000 replications. The viruses sequenced in this study are shown in red in the phylogenetic trees. Scale bars indicate nucleotide substitutions per site.


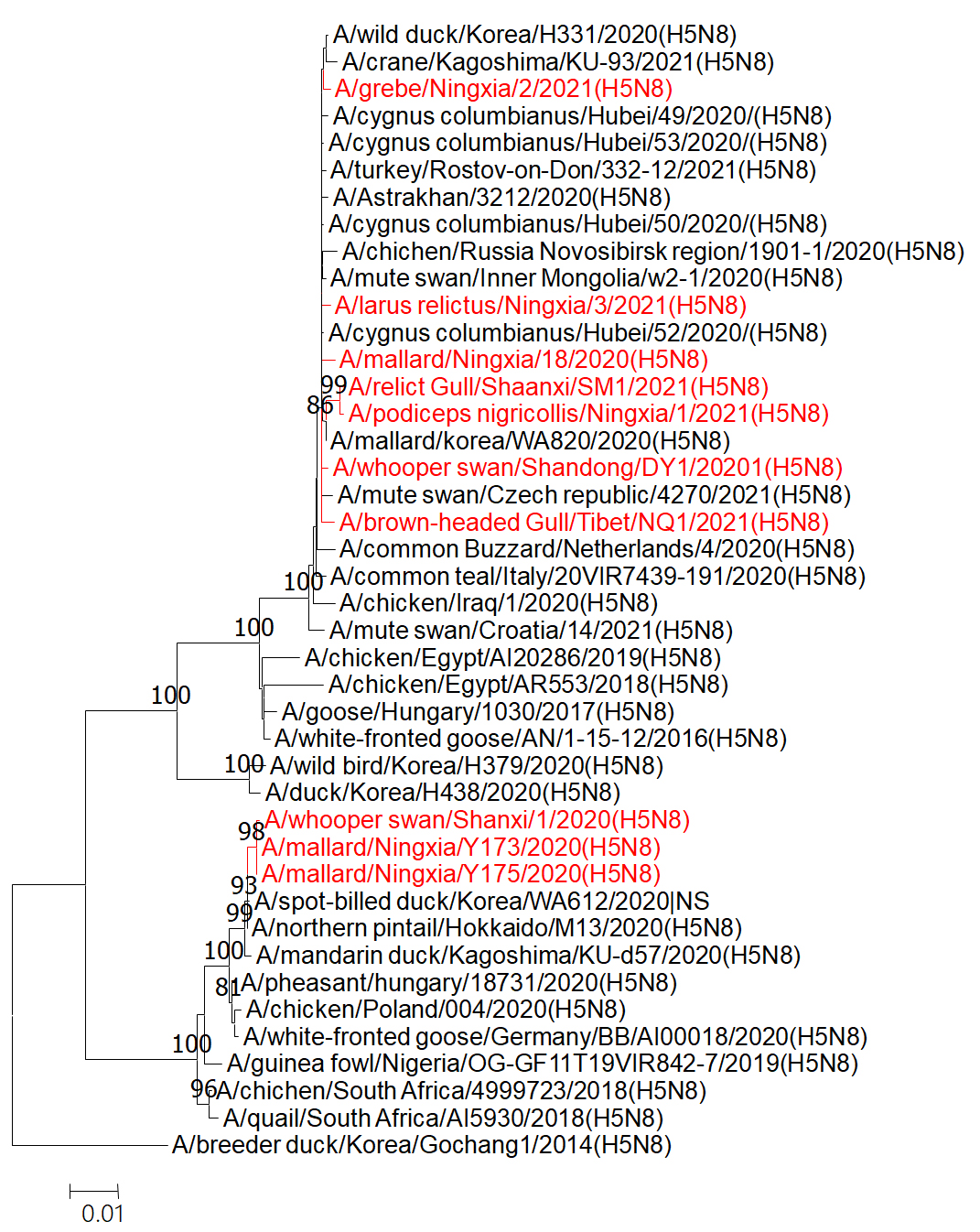


Figures S4. Phylogenetic analyse of the NP gene of H5N8 highly pathogenic avian influenza viruses. Trees were constructed with MEGA5.10 software using the neighbor-joining method. Bootstrap analysis was performed with 1,000 replications. The viruses sequenced in this study are shown in red in the phylogenetic trees. Scale bars indicate nucleotide substitutions per site.


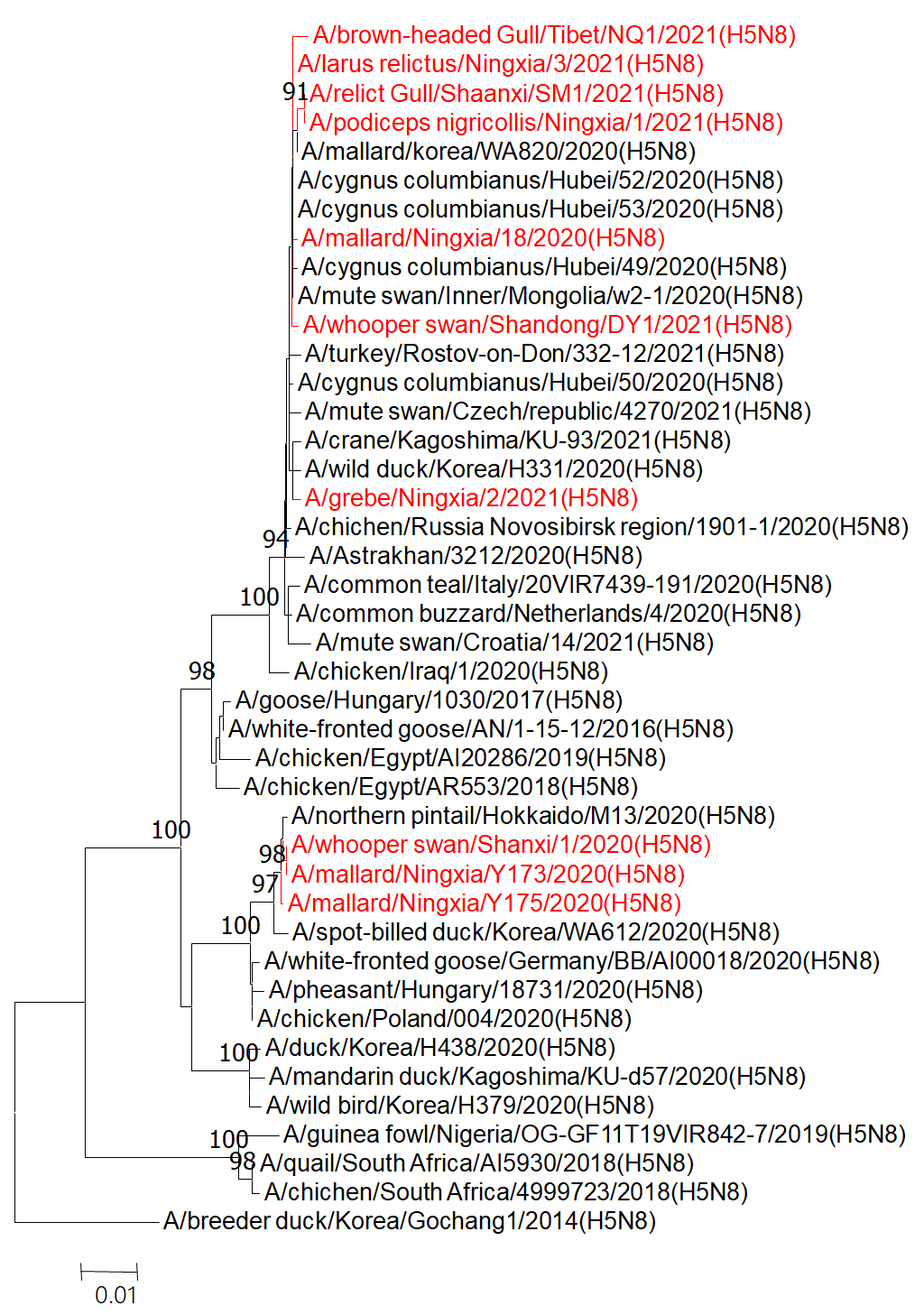


Figures S5. Phylogenetic analyse of the M gene of H5N8 highly pathogenic avian influenza viruses. Trees were constructed with MEGA5.10 software using the neighbor-joining method. Bootstrap analysis was performed with 1,000 replications. The viruses sequenced in this study are shown in red in the phylogenetic trees. Scale bars indicate nucleotide substitutions per site.


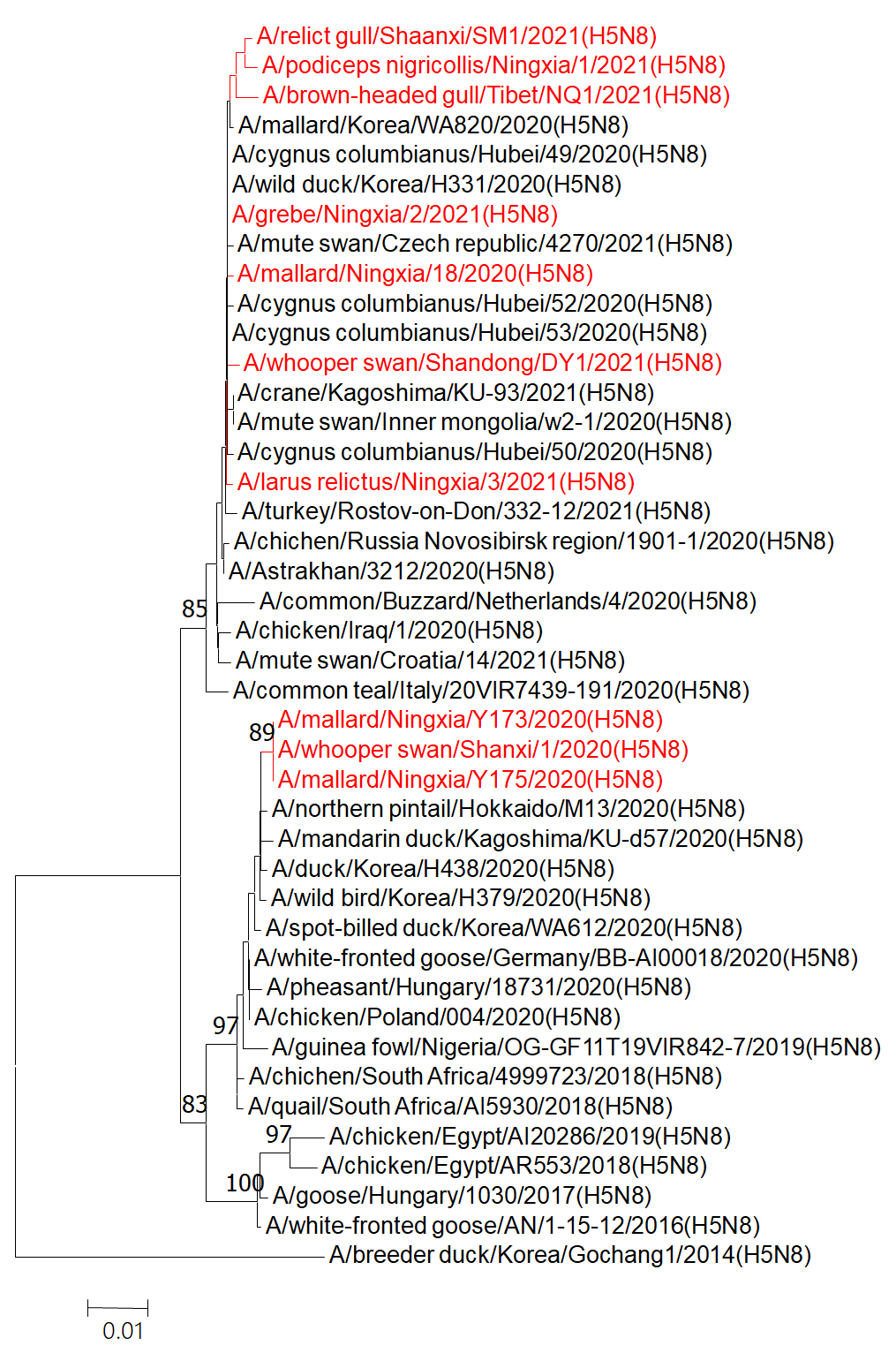


Figures S6. Phylogenetic analyse of the NS gene of H5N8 highly pathogenic avian influenza viruses. Trees were constructed with MEGA5.10 software using the neighbor-joining method. Bootstrap analysis was performed with 1,000 replications. The viruses sequenced in this study are shown in red in the phylogenetic trees. Scale bars indicate nucleotide substitutions per site.


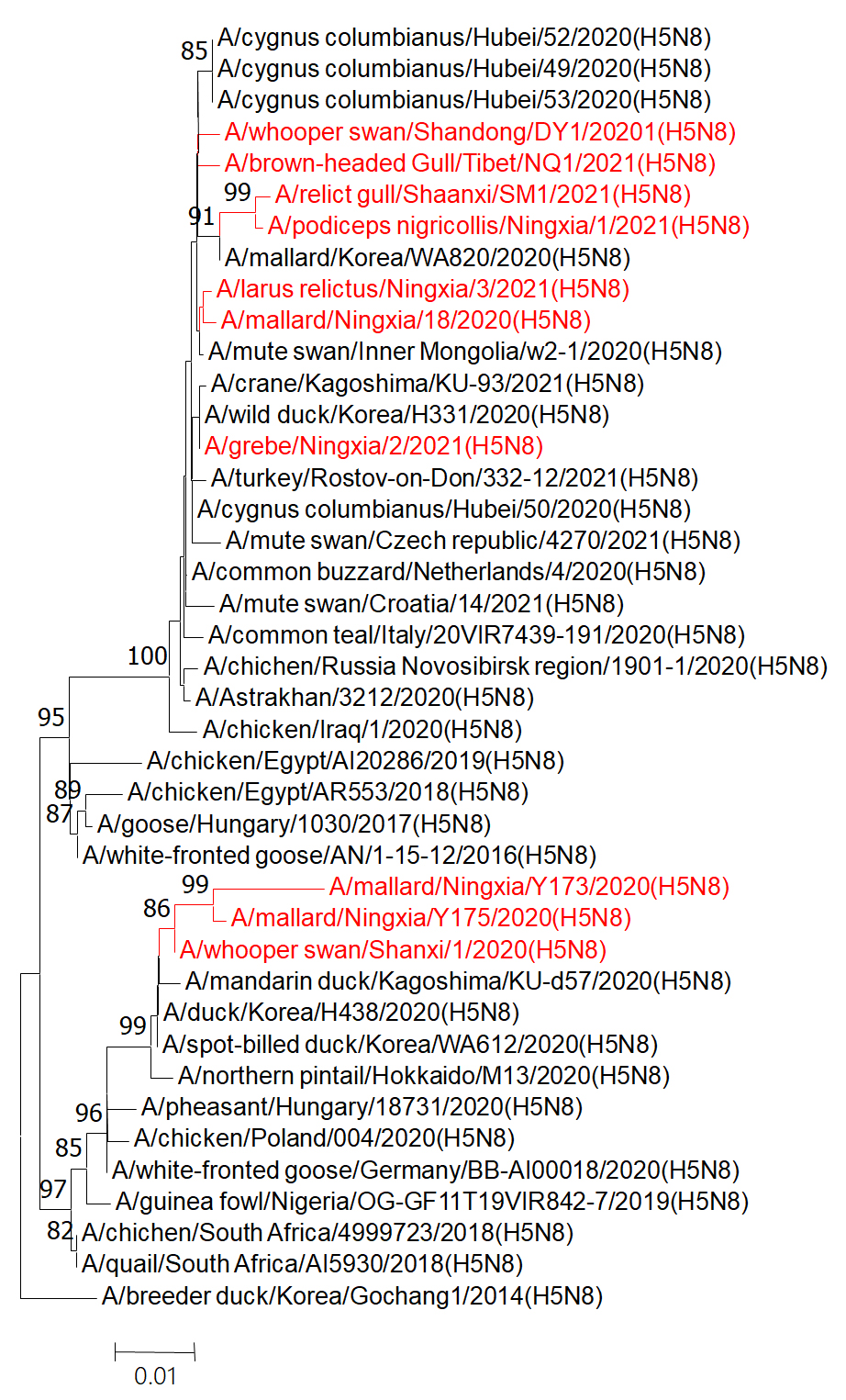


Figures S7. Phylogenetic analyse of the NA gene of H5N8 highly pathogenic avian influenza viruses. Trees were constructed with MEGA5.10 software using the neighbor-joining method. Bootstrap analysis was performed with 1,000 replications. The viruses sequenced in this study are shown in red in the phylogenetic trees. Scale bars indicate nucleotide substitutions per site.


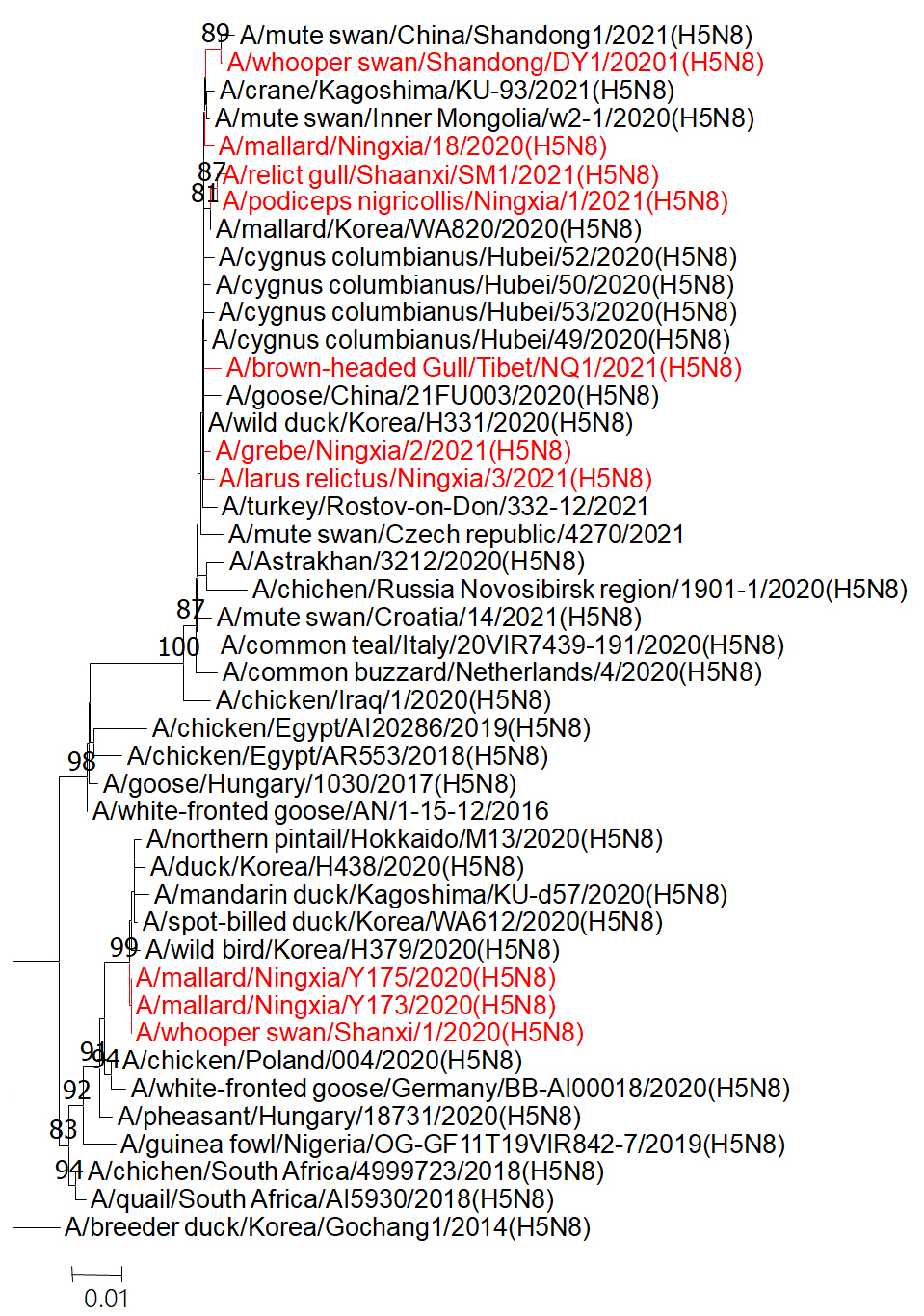


Table S1. The information of the H5N8 viruses isolated from wild birds in 2020-2021, China.

| Strains | Date | Provinces | Abbreviation |
| --- | --- | --- | --- |
| A/whooper swan/Shanxi/1/2020(H5N8) | 2020-11-26 | Shanxi | SX1/2020 |
| A/mallard/Ningxia/18/2020(H5N8) | 2020-11-25 | Ningxia | NX18/2020 |
| A/mallard/Ningxia/Y173/2020(H5N8) | 2020-10-15 | Ningxia | Y173/2020 |
| A/mallard/Ningxia/Y175/2020(H5N8) | 2020-10-15 | Ningxia | Y175/2020 |
| A/whooper swan/Shandong/DY1/20201(H5N8) | 2021-1-19 | Shandong | SY1/2021 |
| A/relict gull/Shaanxi/SM1/2021(H5N8) | 2021-6-9 | Shaanxi | SM1/2021 |
| A/podiceps nigricollis/Ningxia/1/2021(H5N8) | 2021-6-20 | Ningxia | NX1/2021 |
| A/brown-headed gull/Tibet/NQ1/2021(H5N8) | 2021-5-19 | Tibet | NQ1/2021 |
| A/grebe/Ningxia/2/2021(H5N8) | 2021-6-20 | Ningxia | NX2/2021 |
| A/larus relictus/Ningxia/3/2021(H5N8) | 2021-6-20 | Ningxia | NX3/2021 |

Table S2. Antigenic analysis of H5N8 avian influenza viruses by cross-HI tests.

| Antigens | HI titers of antibodies against antigens (log2) | | | | | | | | | | |
| --- | --- | --- | --- | --- | --- | --- | --- | --- | --- | --- | --- |
|  | SX1/2020 | NX18/2020 | Y173/2020 | Y175/2020 | SY1/2021 | SM1/2021 | NX1/2021 | NQ1/2021 | NX2/2021 | NX3/2021 | **Re-11** |
| SX1/2020 | **9** | **9** | **8** | **8** | **9** | **8** | **8** | **9** | **7** | **9** | **4** |
| NX18/2020 | **9** | **9** | **8** | **8** | **8** | **8** | **7** | **8** | **8** | **8** | **4** |
| Y173/2020 | **8** | **8** | **9** | **7** | **8** | **8** | **7** | **9** | **7** | **8** | **5** |
| Y175/2020 | **9** | **8** | **8** | **9** | **8** | **8** | **7** | **8** | **7** | **8** | **5** |
| SY1/2021 | **8** | **7** | **7** | **7** | **9** | **7** | **7** | **7** | **7** | **7** | **5** |
| SM1/2021 | **7** | **7** | **7** | **7** | **7** | **9** | **7** | **7** | **7** | **8** | **3** |
| NX1/2021 | **8** | **7** | **7** | **7** | **7** | **7** | **9** | **7** | **7** | **8** | **3** |
| NQ1/2021 | **9** | **8** | **8** | **8** | **7** | **8** | **8** | **9** | **8** | **9** | **5** |
| NX2/2021 | **7** | **7** | **7** | **7** | **7** | **7** | **7** | **7** | **9** | **8** | **3** |
| NX3/2021 | **8** | **7** | **8** | **8** | **7** | **7** | **7** | **7** | **8** | **9** | **3** |
| **Re-11** | **5** | **4** | **4** | **5** | **5** | **4** | **3** | **4** | **3** | **4** | **9** |
